# Predictors of accurate intrapedicular screw placement in single-level lumbar (L4-5) fusion: robot-assisted pedicle screw, traditional pedicle screw, and cortical bone trajectory screw insertion

**DOI:** 10.1186/s12893-022-01733-6

**Published:** 2022-07-24

**Authors:** Hua-Qing Zhang, Can-Can Wang, Ren-Jie Zhang, Lu-Ping Zhou, Chong-Yu Jia, Peng Ge, Cai-Liang Shen

**Affiliations:** Department of Orthopedics, Spine Surgery, The First Affiliated Hospital of Anhui Medical University, 210 Jixi Road, Hefei, Anhui China

**Keywords:** Proximal facet joint violation, Minimally invasive surgery, Cortical bone trajectory, Robotic‐assisted pedicle screw, Traditional pedicle screw, Screw accuracy

## Abstract

**Background:**

The superiorities in proximal facet joint protection of robot-assisted (RA) pedicle screw placement and screw implantation via the cortical bone trajectory (CBT) have rarely been compared. Moreover, findings on the screw accuracy of both techniques are inconsistent. Therefore, we analyzed the screw accuracy and incidence of facet joint violation (FJV) of RA and CBT screw insertion in the same study and compared them with those of conventional pedicle screw (PS) insertion. The possible factors affecting screw accuracy and FJV were also analyzed.

**Methods:**

A total of 166 patients with lumbar degenerative diseases requiring posterior L4-5 fusion were retrospectively included and divided into the RA, PS, and CBT groups from March 2019 to December 2021. The grades of intrapedicular accuracy and superior FJV were evaluated according to the Gertzbin–Robbins scale and the Babu scale based on postoperative CT. Univariable and multivariable analyses were conducted to assess the possible risk factors associated with intrapedicular accuracy and superior FJV.

**Results:**

The rates of optimal screw insertion in the RA, PS, and CBT groups were 87.3%, 81.3%, and 76.5%, respectively. The difference between the RA and CBT groups was statistically significant (P = 0.004). Superior FJVs occurred in 28.2% of screws in RA, 45.0% in PS, and 21.6% in CBT. The RA and CBT groups had fewer superior FJVs than the PS group (P = 0.008 and P < 0.001, respectively), and no significant difference was observed between the RA and CBT groups (P = 0.267). Multivariable analysis revealed that the CBT technique was an independent risk factor for intrapedicular accuracy. Furthermore, older age, the conventional PS technique and a smaller facet angle were independently associated with the incidence of superior FJVs.

**Conclusions:**

The RA and CBT techniques were associated with fewer proximal FJVs than the PS technique. The RA technique showed a higher rate of intrapedicular accuracy than the CBT technique. The CBT technique was independently associated with screw inaccuracy. Older age, conventional PS technique and coronal orientation of the facet join were independent risk factors for superior FJV.

## Background

Pedicle screw fixation facilitates stable three-column fixation and has become a standard technique for the treatment of various lumbar diseases [1, 2]. Although many studies have reported that the early results of spinal fusion were promising, the long-term clinical efficacy can be compromised because of adjacent segment disease (ASD). ASD has become the major cause of revision surgical procedures after lumbar fusion [3]. Recent studies have shown that cranial facet joint violation (FJV) is an important risk factor for ASD [4–8]. Injury to the superior facet joints during the placement of pedicle screws was associated with alterations in stability and the load-bearing capability of the adjacent segment [9–11], thus accelerating the degeneration of the joint and ultimately leading to ASD. The rates of FJV in different implantation techniques varies. Percutaneous pedicle screw placement was associated with significantly more cranial facet violations than traditional open surgery [12]. Two emerging techniques, robot-assisted (RA) screw placement and screw implantation via the cortical bone trajectory (CBT), have been gradually applied in clinical practice in recent years [13–16]. These two implantation techniques have shown remarkable superiority in proximal facet joint protection [17–20]. However, these two techniques have rarely been compared. Considering the special anatomical proximity of the pedicle, screw malposition can cause serious complications. Hence, screw accuracy is consistently regarded as one of the criteria for the measurement of the quality of each screw implantation technique. Both the RA and CBT screw implantation techniques are emerging minimally invasive techniques that can reduce paravertebral muscle dissection, but findings on the screw accuracy of both techniques are inconsistent [13, 21–27].

Therefore, we analyzed the screw accuracy and incidence of FJVs of RA and CBT screw insertion in the same study and compared them with those of conventional pedicle screw insertion.

## Methods

### Study design and patients

The study was approved by the hospital institutional review board. A consecutive series of lumbar fusions performed between March 2019 and December 2021 were retrospectively reviewed. Patients were selected using the following inclusion criteria: age between 20 and 80 years old; inclusion for single-level fusion of L4-5 due to lumbar degenerative disease associated with segmental instability, including huge disc herniation, lumbar spinal stenosis, and spondylolisthesis (grade I/II); ineffective results with conservative treatment for at least 6 months before surgery; and postoperative CT examination. The exclusion criteria were as follows: decompression without fusion; previous surgery on the lumbar spine; congenital malformations of the lumbar spine; and incomplete imaging data. All patients underwent surgery by the same team of experienced surgeons. The choice of insertion approach was based on a discussion between the surgeon and the patient and was related to inadequate reimbursement for use of RA in many cases.

## Surgical techniques

### RA pedicle screw placement

In the RA group, according to the intraoperative 3D fluoroscopic images of the surgical area, the surgeon planned screw trajectories on the robotic workstation (TINAVI, China). After the robotic arm moving to the planned path, the placement of 4 guide pins were completed through the guidance of robotic arm. The guide pins were fixed properly, then an interbody polyetheretherketone (PEEK) cage was placed under the tubular retractors. After decompression, adequate-sized cannulated screws were subsequently inserted.

### Traditional PS placement

In the PS group, a midline incision was made, followed by exposure of the spine to the spinous processes, laminae, and facet joint to allow clear identification of the bony landmarks. The entry point and trajectory were confirmed fluoroscopically. Adequate-sized pedicle screws were subsequently inserted. After decompression, an interbody PEEK cage was placed, and the rods and screw caps were inserted.

### CBT screw placement

Through a midline incision, subperiosteal dissection was performed down to the spinous processes, laminae, and facet joint. The entry point of the cortical screw was located at the center of the superior articular process and 1 mm inferior to the inferior border of the transverse process. The trajectory direction was 10° laterally in the axial plane and 25°–30° cranially in the sagittal plane. The entry point and trajectory were determined according to the C-arm perspective. The initial hole was made using a 2.0-mm high-speed burr drill. The hole was deepened to 35 mm by using a 2.5-mm hand drill and subsequently tapped to 4.5 mm. After PLIF, adequate-sized screws and rods were inserted.

### Outcome measure

The following imaging indicators were measured based on the postoperative CT scans.

### Intrapedicular accuracy

**Fig. 1:**
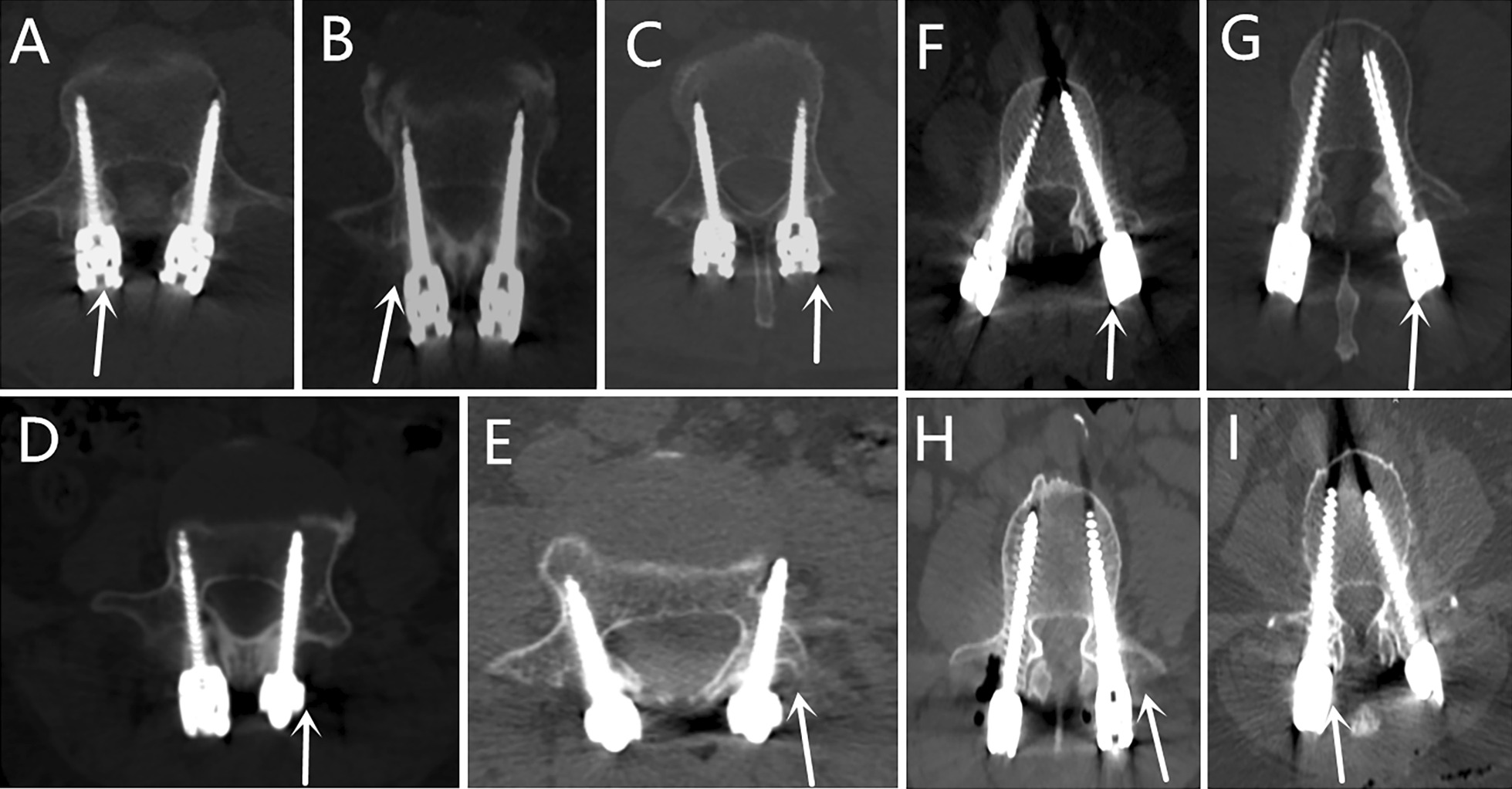
Grades of intrapedicular accuracy and facet joint violation: **A** grade A, **B** grade B, **C** grade C, **D** grade D, and **E** grade E; **F** Grade 0, **G** Grade 1, **H** Grade 2, and **I** Grade 3

Screw accuracy was evaluated according to the Gertzbin and Robbins scale [28] (Fig. 1). Grade A (no cortical penetration); grade B (the distance of cortical penetration ≤ 2 mm); grade C (2 mm ≤ the distance of cortical penetration < 4 mm); grade D (4 mm ≤ the distance of cortical penetration < 6 mm); grade E (the distance of cortical penetration ≥ 6 mm). Grade A was regarded as the excellent position, grades A and B indicated clinically acceptable positions, while grades C, D, and E were considered clinically unacceptable positions.

### Screw violation grade

The grade of proximal FJV was determined in accordance with the Babu scale [29] (Fig. 1) and was classified as grade 0 (screw not in facet), grade 1 (screw in lateral facet but not in facet articulation), grade 2 (penetration of facet articulation less than 1 mm), and grade 3 (penetration of facet articulation more than 1 mm or traveling within the facet articulation).

**Fig. 2:**
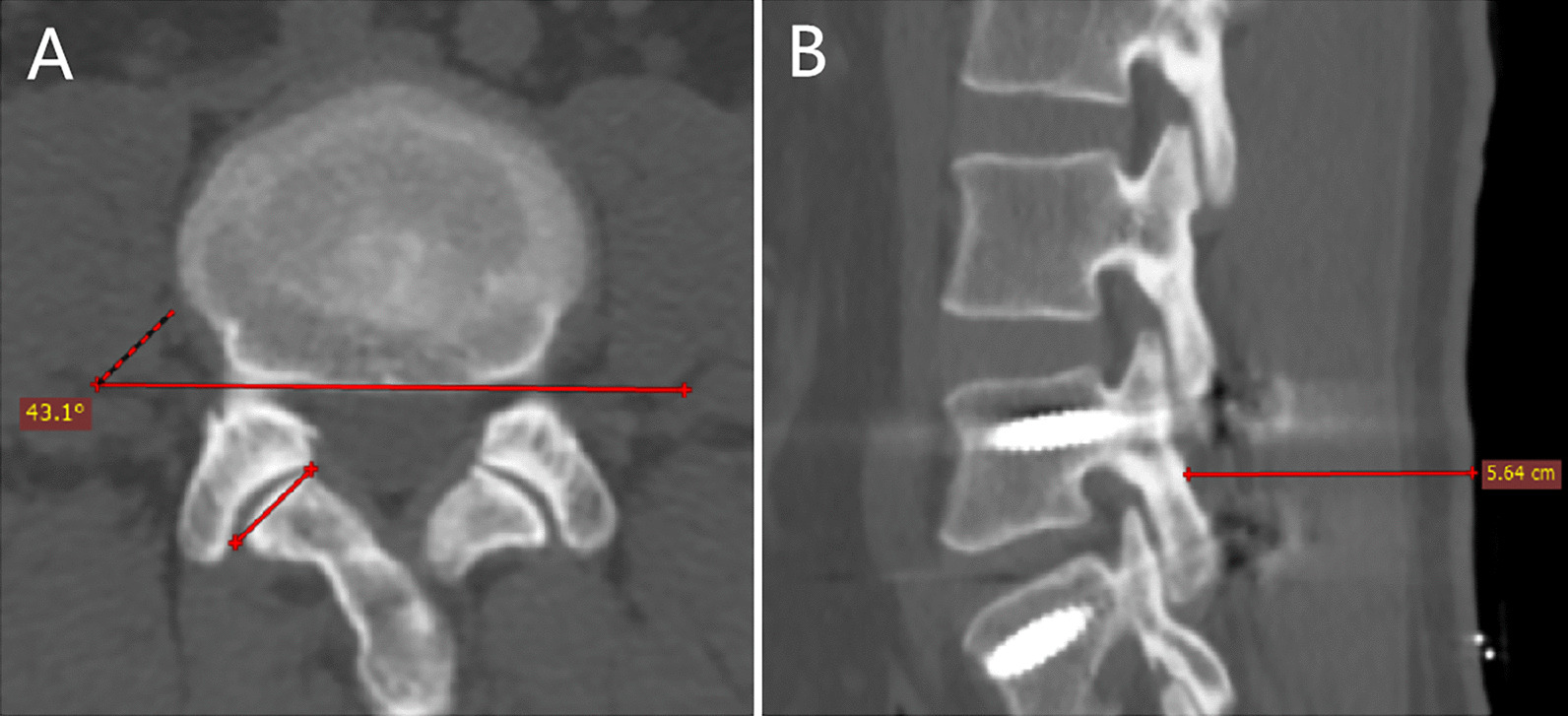
Measurement of superior facet angle (**A**) and incision depth (**B**)

Other variables were also measured during assessment of the postoperative CT scan, such as the incision depth (defined as the distance between the L4 lamina and skin), superior facet angle (measured as described by Grobler et al. [30], Fig. 2) and the degree of L4 vertebral slippage. Radiographic data were independently measured by two spinal surgeons, who had professionally mastered the measurement methods for screw accuracy, detection of proximal FJV and related indicators. If divergences existed between the two evaluators, a third evaluator made the final decision. As secondary parameters, we recorded the surgical time from skin to skin (min), intraoperative blood loss (mL), postoperative drainage (mL), drop in hemoglobin after surgery (g/L), postoperative hospital stay (days), and perioperative complications (e.g. dural tear, wound infections, intraoperative revision caused by screw malposition and neurologic deficit). The preoperative diagnosis, age, sex, BMI, VAS score for back pain, and VAS score for leg pain were also noted for each patient.

### Statistical analysis

Statistical analyses were performed using SPSS 25.0 software (IBM Corp., Armonk, New York, USA). Continuous variables were presented as the mean and standard deviation. Analysis of variance was carried out to compare variables among the three groups and the least significant difference (LSD) method was used for subsequent pairwise comparisons. P < 0.05 was considered statistically significant. The rank-sum test was used under the condition of heterogeneity of variance, and the post hoc Bonferroni correction for multiple comparisons was applied (significance level adjusted to 0.0167). Categorical variables were expressed as absolute (no.) and relative (%) frequencies, and the chi-square test was used for analysis. Interobserver reliabilities for screw accuracy and FJV assessment were measured with the weight kappa coefficient. The reliabilities were high (κ = 0.867 and 0.892, respectively), indicating that the measurements were reliable. The factors that affected the intrapedicular accuracy and proximal FJV were explored through univariable analysis in terms of sex, age, BMI, superior facet angle, implantation technique, vertebral slip more than 10%, and incision depth. Binary logistic regression with the enter method was used, and the final model maintained only the predictors with a significance level < 0.05 during the univariable analysis (if there were few influencing factors, the inclusion level was relaxed to 0.1). All findings were reported in accordance with the Strengthening the Reporting of Observational Studies in Epidemiology (STROBE) guidelines [31].

## Results

### Demographic data

CTs were unavailable in 14 patients (7.8%) due to refusal. A total of 166 patients were included in the present study, including 65 males and 101 females. Among them, 55 patients underwent RA screw insertion, 60 patients underwent PS screw insertion, and 51 patients underwent CBT screw insertion. No significant differences were observed in terms of sex, age, body mass index (BMI), preoperative diagnosis, or preoperative pain score (P > 0.05) (Table 1).

**Table 1:** Descriptive statistics of the included patients in the trial

Characteristics	No. of patients			P value
	RA (*n* = 55)	PS (*n* = 60)	CBT (*n* = 51)	
Sex (male/female)	24/31	23/37	18/33	0.670
Age (years)	54.89 ± 10.67	55.53 ± 10.43	57.08 ± 9.70	0.535
BMI (kg/m^2^)	24.34 ± 2.47	25.05 ± 3.33	24.42 ± 3.24	0.387
Pre-operative diagnosis (*n*)				0.295
LDH	35	36	28	
LCS	10	10	16	
SPL	10	14	7	
Back pain VAS	4.58 ± 1.20	4.68 ± 1.46	4.59 ± 1.50	0.908
Leg pain VAS	5.35 ± 1.39	5.33 ± 1.34	5.59 ± 1.17	0.527

*RA* robot-assisted, *PS* pedicle screw, *CBT* cortical bone trajectory, *BMI* body mass index, *LDH* lumbar discal hernia, *LCS* lumbar canal stenosis, *SPL* spondylolisthesis, *VAS* visual analogue scale

### Perioperative data

The drop in hemoglobin after surgery and the incidence of adverse events were not significantly different among the three groups (P = 0.788, P = 0.313, Table 2). Compared to the other two groups, the RA group had a lower intraoperative blood loss, postoperative drainage, and postoperative hospital stay but had a longer surgical time. Meanwhile, the CBT group had a similar postoperative drainage and postoperative hospital stay but lower intraoperative blood loss and a longer surgical time than the PS group (Table 3).

**Table 2:** Perioperative data of the included patients in the trial

Valuables	RA (*n* = 55)	PS (*n* = 60)	CBT (*n* = 51)	P value
Surgical time from skin to skin (min)	167.51 ± 29.60	121.12 ± 34.29	138.63 ± 34.24	< 0.001
Intra-operative blood loss (mL)	201.82 ± 64.52	305.00 ± 81.15	242.16 ± 72.37	< 0.001
Post-operative drainage (mL)	8.10 ± 37.74	336.42 ± 220.22	263.14 ± 185.14	< 0.001
Drop of HGB after surgery (g/L)	20.40 ± 11.55	18.58 ± 9.99	17.67 ± 8.81	0.372
Post-operative hospital stay (days)	4.0 ± 0.90	5.57 ± 2.90	5.43 ± 1.85	< 0.001
Adverse events (*n*)	1 (1.8%)	5 (8.4%)	4 (7.9%)	0.307
Dural tears	0	1 (1.7%)	1 (2.0%)	
Wound infections	0	3 (5%)	1 (2.0%)	
Intra-operative revision caused by screw malposition	1 (1.8%)	0	2 (3.9%)	
Neurologic deficit	0	1 (1.7%)	0	

*RA* robot-assisted, *PS* pedicle screw, *CBT* cortical bone trajectory, *LDH* lumbar discal hernia, *LCS* lumbar canal stenosis, *SPL* spondylolisthesis, *HGB* hemoglobin

**Table 3:** Pairwise analysis of perioperative data

	Group	Standard error	P
Surgical time from skin to skin	RA and PS	6.122	< 0.001*
	RA and CBT	6.375	< 0.001
	PS and CBT	6.246	0.006
Intra-operative blood loss (mL)	RA and PS	8.062	< 0.001†
	RA and CBT	8.396	0.006
	PS and CBT	8.226	< 0.001
Post-operative drainage (mL)	RA and PS	8.832	< 0.001†
	RA and CBT	9.197	< 0.001
	PS and CBT	9.011	0.075
Post-operative hospital stay (days)	RA and PS	0.390	< 0.001*
	RA and CBT	0.406	0.001
	PS and CBT	0.398	0.734

*RA* robot-assisted, *PS* pedicle screw, *CBT* cortical bone trajectory

P < 0.05 indicates that statistical differences were observed between the two groups

^*^Analysis of variance, LSD for posterior comparison: †Kruskal–Wallis H test of rank test, and Bonferroni for posterior comparison. The significance level in the table has been adjusted for multinomial tests

### Screw accuracy

The rates of optimal screw (Grade A) in the RA, PS, and CBT groups were 87.3%, 81.3%, and 76.5%, respectively (Table 4). Pairwise comparisons revealed that the difference between the RA and CBT groups was statistically significant (P = 0.004) (Table 5). In addition, the rates of clinically acceptable screw (Grade A + B) were 98.2%, 97.5%, and 94.1%, indicating no significant differences among the three techniques (P = 0.616, 0.028, 0.072) (Table 5).

**Table 4:** Comparison of screw accuracy and superior FJVs in three different insertion techniques

Parameters	Number of screws			P value
	RA (*n* = 55)	PS (*n* = 60)	CBT (*n* = 51)	
*Screw grade*				0.012
A	192 (87.3%)	195 (81.3%)	156 (76.5%)	
B	24 (10.9%)	39 (16.2%)	36 (17.6%)	
C	4 (1.8%)	6 (2.5%)	10 (4.9%)	
D	0 (0)	0 (0)	1 (0.5%)	
E	0 (0)	0 (0)	1 (0.5%)	
A + B	216 (98.2%)	234 (97.5%)	192 (94.1%)	0.045
C + D + E	4 (1.8%)	6 (2.5%)	12 (5.9%)	
*Violation grade*				0.001
0	79 (71.8%)	66 (55.0%)	80 (78.4%)	
1	23 (20.9%)	51 (42.5%)	22 (21.6%)	
2	7 (6.4%)	3 (2.5%)	0 (0)	
3	1 (0.9%)	0 (0)	0 (0)	

*RA* robot-assisted, *PS* pedicle screw, *CBT* cortical bone trajectory, *FJV* facet joint violation

**Table 5:** Pairwise analysis of the rates of the optimal screw, the clinically acceptable screw and the rates of FJV

	Group	^χ2^	P*
Pairwise analysis of the rates of the optimal screw	RA and PS	3.118	0.077
	RA and CBT	8.395	0.004
	PS and CBT	1.521	0.217
Pairwise analysis of the rates of the clinically acceptable screw	RA and PS	0.251	0.616
	RA and CBT	4.815	0.028
	PS and CBT	3.243	0.072
Pairwise analysis of the rates of facet violation	RA and PS	6.967	0.008
	RA and CBT	1.234	0.267
	PS and CBT	13.445	< 0.001

*RA* robot-assisted, *PS* pedicle screw, *CBT* cortical bone trajectory, *FJV* facet joint violation

^*^P < 0.017 indicates that statistical differences were observed between the two groups

### Superior FJV

Superior FJVs occurred in 28.2% of screws in the RA group and 21.6% of screws in the CBT group. In the PS group, the rate reached 45%. The RA and CBT groups had fewer superior FJVs than the PS group (P = 0.008 and P < 0.001, respectively), and no significant difference was observed between the RA and CBT groups (P = 0.267) (Table 5).

### Factors associated with intrapedicular accuracy and FJV

CBT screw technique, BMI and incision depth were included in the final logistic regression analysis model of intrapedicular accuracy (P < 0.1) (Table 6). The results demonstrated that the CBT screw technique was an independent risk factor for intrapedicular accuracy (odds ratio [OR] = 3.527, P = 0.007) (Table 7). Age, the conventional PS technique, superior facet angle and pedicle cortex penetration were associated with FJVs in the univariable analysis (P < 0.05, Table 8). Binary logistic regression analysis was conducted with these variables, and the following independent factors for FJV were identified: superior facet angle (OR = 0.931, P < 0.001), PS (OR = 3.508, P < 0.001), and age (OR = 1.039, P = 0.003) (Table 9).

**Table 6:** Univariate analyses of factors associated with intrapedicular accuracy

Risk factors	Clinical accepted screw group	Clinical unacceptable screw group	P-value
No. of screws	644	20	
Age	55.8 ± 10.1	56.4 ± 13.9	0.789
Sex			0.188
Male	255	5	
Female	389	15	
BMI	24.7 ± 3.0	23.4 ± 2.7	0.064
Implantation technique			0.004*
RA + PS	452	8	
CBT	192	12	
Vertebral slip more than 10%			0.957
Yes	132	4	
No	512	16	
Incision depth	55.6 ± 8.0	52.1 ± 7.9	0.062
Superior facet angle	48.0 ± 13.0	44.8 ± 11.9	0.286

*RA* robot-assisted, *PS* pedicle screw, *CBT* cortical bone trajectory, *BMI* body mass index, *FJV* facet joint violation

**Table 7:** Logistic regression model of variables associated with intrapedicular accuracy

Risk factors	B value	p-value	Adjusted OR	95% CI
CBT	1.260	0.007	3.527	1.410–8.824
BMI	− 0.085	0.316	0.919	0.788–1.084
Incision depth	− 0.050	0.139	0.951	0.890–1.016

*CBT* cortical bone trajectory, *BMI* body mass index, *CI* confidence interval

**Table 8:** Univariate analyses of factors associated with superior FJV

Risk factors	FJV group	Non-FJV group	P-value
No. of screws	107 (32.2%)	225 (67.8%)	
Age	58.03 ± 10.37	54.73 ± 10.05	0.006
Sex			0.051
Male	50 (46.7%)	80 (35.6%)	
Female	57 (53.3%)	145 (64.4%)	
BMI	24.60 ± 2.94	24.63 ± 3.09	0.922
Implantation technique			0.001
RA + CBT	53 (25%)	159 (75%)	
PS	54 (45%)	66 (55.0%)	
Vertebral slip more than 10%			0.369
Yes	25 (23.4%)	43 (19.1%)	
No	82 (76.6%)	182 (80.9%)	
Incision depth	55.35 ± 7.49	55.63 ± 8.31	0.769
Superior facet angle	48.64 ± 8.94	53.63 ± 9.82	< 0.001
Pedicle cortex			0.048
Intact	74	178	
Breached	33	47	

*RA* robot-assisted, *PS* pedicle screw, *CBT* cortical bone trajectory, *BMI* body mass index, *FJV* facet joint violation

**Table 9:** Logistic regression model of variables associated with superior facet violation

Risk factors	B value	p-value	Adjusted OR	95% CI
Age	0.038	0.003	1.039	1.013–1.065
PS	1.255	< 0.001	3.508	2.058–5.980
Superior facet angle	− 0.072	< 0.001	0.931	0.904–0.958
Pedicle cortex penetration	0.492	0.093	1.636	0.921–2.905

*PS* pedicle screw, *CI* confidence interval

## Discussion

As lumbar fusion has become increasingly used in clinical practice, the incidence of ASD, one of the late complications of this procedure, has also increased gradually [7]. Superior FJV, as an important risk factor for ASD [6–8], has attracted increasing attention. Since screw malposition may cause serious complications [32], the safety and accuracy of screw placement are critical. Thus, different insertion techniques have been applied to increase intrapedicular accuracy and reduce the incidence of superior FJVs [13, 19, 24]. The RA and CBT techniques are novel screw placement methods that have been reported to be associated with a low incidence of FJVs [18, 19, 33, 34]. However, the FJV incidence of these two techniques has not been compared. In terms of screw accuracy, the literature reporting on these two techniques has not been consistent [21, 23, 25, 27]. Therefore, we analyzed the screw accuracy and FJV incidence of RA and CBT in the same study and compared them with those of conventional PS insertion.

### Screw accuracy and risk factors

The combined results of the intrapedicular accuracy of RA screw placement in previously published literatures are not consistent. Kim et al. [24] found no remarkable difference between RA (Renaissance, Israel) and freehand pedicle screw insertion. By using the same robot navigation system, Molliqaj et al. [23] found that RA screw placement had higher accuracy than the freehand technique (93.4% vs. 88.9%), while Ringel et al. [21] found that RA screws were less accurate than conventional screws (85% vs. 93%), and most malpositioned RA screws were laterally deviated (SpineAssist, Israel). In the present study, we used the TiRobot system (TINAVI Medical Technologies, Beijing, China). TINAVI robot-assisted screw placement can remarkably improve the precision of screws [13, 35]. The results of the present study show that the RA screws had the highest accuracy (98.3% clinically acceptable screws), no grade D and E accuracies, no screw-related complications, and high surgical safety.

Few studies have focused on assessing the insertion accuracy for CBT screws. Thus, there is no specific accuracy criterion for CBT screws. However, some researchers have applied the evaluation methodology of traditional pedicle screws to CBT screws [25, 36]. Tan et al. [27] used 3D imaging and visualization software to study the safety of freehand CBT techniques and found that 78% of the screws had ideal and safety trajectories, while the remaining 22% were unsafe. Ishii et al. [25] studied the accuracy of freehand CBT screw insertion and found that 3.3% were unacceptable, while 2.2% required revision. Based on their experience in CBT screw placement with more than 20 human cadavers for biomechanical study, the operator in our study used bone anatomical landmarks combined with intraoperative fluoroscopy. The results showed 94.1% acceptable screw insertion for CBT, a 5.9% unacceptable screw rate, and a 7.9% perioperative complication rate with no serious complications that required reoperation. The screw accuracy of the CBT technique in our study was high but still lower than that of the other two techniques. Multivariable analyses revealed that the CBT screw technique is an independent risk factor for screw accuracy. Given that the CBT technique was developed in the last 10 years, it has been applied far less often than the conventional PS procedure. Additional experience and improvements in the technique could further increase the screw placement accuracy of this procedure.

### Superior FJV and risk factors

The current study demonstrated that the RA and CBT techniques can reduce the incidence of FJVs, and no substantial difference was observed between these two groups. The lower rate of FJVs in RA screw placement can be explained by the mechanism of guidance. The TiRobot system used in this study can choose the entry point and trajectory of the screws during planning. When facet joint invasion is observed on the blueprint, we can move the entry point outside, increase the screw tilt angle, and effectively avoid an FJV. This phenomenon is difficult to actualize in conventional open surgery, because the outward migration of the entry point needs to overcome the resistance of the paravertebral soft tissues and requires wide muscle dissection. Similarly, the CBT screw technique can effectively reduce the FJV rate because the entry point of the CBT screw is near the pars articularis, which is far from the superior facet joint, and because of the downsizing of the screw diameter (4.5–5.5 mm), and this configuration can reduce the probability of joint damage.

With a high FJV rate, the conventional PS insertion technique was an independent risk factor for FJVs in our study. Chen et al. [37] and Chung et al. [38] studied the incidence of FJVs in several commonly used insertion methods for conventional PS and found that the lowest incidence of FJVs was achieved with the intersection technique, followed by the Weinstein technique and the mamillary process technique, whereas the Roy-Camille method had the highest incidence, reaching up to 100%. Considering that the Roy-Camille method requires removal of the tip of the superior articular process, the screw invades the joint when it is screwed into the tail touching the facet joint. However, patients with serious degeneration, unclear anatomical landmarks, and high soft tissue tenson are very common in clinical practice, and we are obliged to move inside the entry point, remove the tip of the superior articular process, and pass through the exposed spongy bone into the pedicle. Under these conditions, the RA and CBT techniques are recommended.

In addition, the present study revealed that a smaller facet angle and older age were significant risk factors independent of the surgical technique. Teles et al. [39] and Patel et al. [40] compared the incidence of FJVs between percutaneous and open screw placement and found that facet angle was an independent risk factor for FJVs. The morphology of the facet joint varies greatly among individuals. The direction of the facet joint from L1 to L5 gradually decreases from the sagittal position to the coronal position [41]. Under a smaller facet angle, the facet joint tends to the coronal position, making it more likely for the screw to invade the facet joint regardless of the technique of screw placement [19].

The effect of age on FJV has not been fully elucidated. Zeng et al. [42] and Patel et al. [40] found that age < 60 years was an independent risk factor for FJV during percutaneous and open screw placement. However, Matsukawa et al. [43] found that age > 70 years was a risk factor for FJV for CBT screws. Similar to the findings of Matsukawa et al., older age in the present study was an independent risk factor for the occurrence of FJVs. The occurrence of FJVs is multifactorial. Young patients have developed paravertebral muscle and high soft tissue tension, thus affecting screw implantation [40, 42]. Severe degeneration of the facet joint in elderly patients makes the choice of entry point difficult and increases the risk of FJVs [43]. During RA screw placement, the entry point is selected according to the planned path. The guide is tension-free, which greatly reduces the impact of the paravertebral soft tissue on the screw placement. The trajectory of CBT screws diverges, and the process of screw placement is less affected by the paravertebral muscles. Therefore, age is an independent risk factor for FJV. This phenomenon also reflects the advantages of the RA and CBT techniques for young patients with developed paravertebral muscle. Therefore, the use of an appropriate implantation technique according to the situation of the patient can reduce the occurrence of FJVs.

### Perioperative data

The RA group had the lowest intraoperative blood loss, postoperative drainage, and postoperative hospital stay, but had the longest surgical time, which is consistent with previous studies [35, 36]. The long surgical time of RA screw placement is attributed to greater intraoperative preparation and the learning curve effect [13]. Lower intraoperative blood loss and a longer surgical time were observed in the CBT group compared with the PS group. Owing to the reduced muscle dissection and shorter incision length, the CBT screw technique can reduce intraoperative blood loss [37]. The longer surgical time is possibly due to the surgeon’s lack of familiarity with the technique [38]. The operation time can be further shortened owing to the potential time saved by the more limited exposure required for CBT screw placement.

The data of the 16 patients in the CBT group were used in our previous article [19], in which screw accuracy and FJV incidence between the CBT and PS placement techniques were compared. In the present study, we increased the number of patients and added the RA group. The superiority of the RA and CBT procedures in superior facet joint protection was compared, that never been reported in previous studies.

We recognize that our study has several limitations. First, although no significant difference was found in the general data of the three groups, a possible bias still existed because this was not a randomized control trial. Second, not all patients had postoperative CT scans (14 patients (7.8%) refused the CT scan because of concerns about radiation damage), which may have biased the results. Third, the clinical effects among the three insertion techniques were not reported in the current study, and the relationship between the different grades of violation and clinical outcomes needs further investigation in future work. Prospective trials with large sample sizes and high quality are also needed in the future.

## Conclusions

The RA and CBT techniques were associated with fewer proximal FJVs than the PS technique. The RA technique showed a higher rate of intrapedicular accuracy than the CBT technique. The CBT technique was independently associated with screw inaccuracy. Older age, conventional PS technique and coronal orientation of the facet join were independent risk factors for superior FJV.

## Data Availability

The datasets used and/or analyzed during the current study are available from the corresponding author on reasonable request.

## Abbreviations

ASD: Adjacent segment degeneration
BMI: Body mass index
CBT: Cortical bone trajectory screw
CT: Computed tomography
FJV: Facet joint violation
LCS: Lumbar canal stenosis
LDH: Lumbar discal hernia
MIS-TLIF: Minimal invasive transforaminal lumbar interbody fusion
PEEK: Polyetheretherketone
PS: Pedicle screw
RA: Robot-assisted
SPL: Spondylolisthesis
PLIF: Posterior lumbar interbody fusion
VAS: Visual analogue scale/score
